# Asymptomatic paroxysmal ventricular standstill in an elderly male following revascularization of multivessel coronary disease: A case report and literature review

**DOI:** 10.1002/ccr3.8675

**Published:** 2024-03-21

**Authors:** Andrew Sagalov, Shashi Maryala, Abhishek Kulkarni, Shruti Hegde, Mohamed Labedi

**Affiliations:** ^1^ SIU School of Medicine, Department of Internal Medicine Springfield Illinois USA; ^2^ SIU School of Medicine Division of Cardiology Springfield Illinois USA

**Keywords:** cardiology, critical care medicine

## Abstract

Ventricular standstill is a dangerous arrhythmia that requires prompt diagnosis and intervention, especially in patients with structural heart disease. Clinicians should recognize ventricular standstill as a complication of cardiac revascularization and be cognizant of asymptomatic cases necessitating intervention. Early evaluation to facilitate pacemaker implantation portends good outcomes in this patient subgroup.

## 1 INTRODUCTION

Ventricular standstill is an uncommon arrhythmia that typically manifests with cardiac arrest. Provoked by interruption of ventricular conduction, early diagnosis is challenging due to most cardiac arrests occurring outside of the hospital. This arrhythmia has been detected in a broad range of clinical scenarios including fulminant myocarditis and as a complication of repair of congenital heart disease. We present an atypical case of asymptomatic ventricular standstill that developed following coronary artery bypass grafting in an elderly male with multivessel coronary disease.

## 2 HISTORY OF PRESENTATION

A 70‐year‐old male presented for elective coronary artery bypass grafting (CABG) after left heart catheterization showed triple vessel disease not amenable to percutaneous coronary intervention (PCI) and multiple in‐stent restenosis (Video S1). In the 3 months prior to admission, he developed progressively worsening chest pain and exertional dyspnea, which prompted the ischemia evaluation. He underwent a nuclear stress test, which was positive for anterior and anterolateral reversible defects. His rest left ventricular ejection fraction (LVEF) was 47% and on stress there was a drop in his LVEF to 31% in addition to a significant drop in blood pressure from 125/77 to 106/44 mmHg. Limited echocardiogram demonstrated a mildly LVEF of 40%–45% and hypokinetic changes in the anterior and lateral walls (Video S2). On admission, he was afebrile to 36.7°C with a heart rate of 87 beats/min and a blood pressure of 130/84 mm Hg. His physical exam was largely unremarkable, including sinus rhythm without murmurs and clear lungs to auscultation. He was breathing comfortably on room air and did not have baseline supplementary oxygen requirements before admission.

## 3 PAST MEDICAL HISTORY

This patient had a past medical history of obstructive coronary disease with multiple stents, hypertension, and Type 2 diabetes mellitus. He did not have any cardiac surgeries or arrhythmia diagnoses prior to this admission. At baseline, he was able to walk at a steady pace without developing cardiac and respiratory symptoms. He had a metabolic equivalent score (METS) of four and was in stable cardiovascular health since his last angiogram.

## 4 INVESTIGATIONS

**FIGURE 1:**
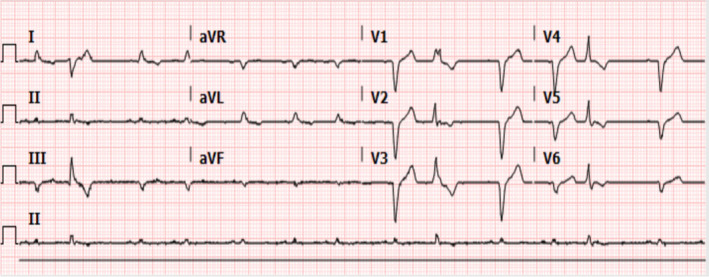
Admission electrocardiogram showing normal sinus rhythm with multiple premature ventricular contractions in anterior leads. An atypical left bundle branch block is visible in V1–V3.

**FIGURE 2:**
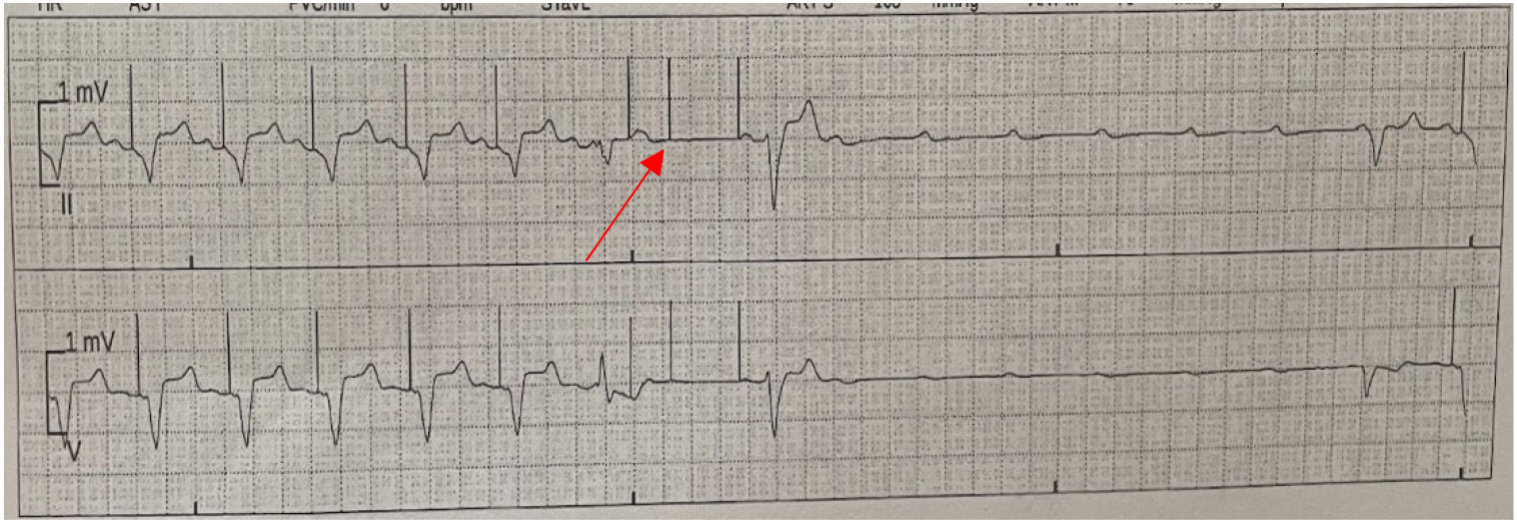
Postoperative day 1 rhythm strip showing a 3–4 s run of nonsustained ventricular standstill in lead II. Pacer spikes from epicardial pacer wires are noted followed by a premature pacer spike, indicating undersensing of the pacemaker (red arrow).

The initial electrocardiogram (EKG) showed sinus rhythm with frequent premature ventricular contractions and an atypical left bundle branch block (Figure 1). Cardiothoracic surgery performed a triple vessel CABG to left anterior descending (LAD), first branch of obtuse marginal (OM1), and first right posterolateral artery (RPL1) in addition to endarterectomy of the middle RPL1. The left internal mammary artery and saphenous vein were used to construct the grafts. For the entirety of the procedure, the patient was maintained on cardiopulmonary bypass and cooled to 34°C with cardioplegia administered every 15–20 min. Cardioplegia served to reduce myocardial oxygen demand in order to minimize the effects of ischemia during the surgery. Due to his obesity, the decision was made to perform sternal plating rather than wire cerclage for sternal reapproximation. Plate fixation is preferred in patients with advanced age and multiple comorbidities since they are at greater risk of developing sternal nonunion and infection postoperatively compared to wire cerclage.^1^ There were no immediate complications from the surgery, and the patient was transferred to the cardiac intensive care unit with temporary epicardial pacing wires. He briefly required hemodynamic support with peripheral norepinephrine, which was quickly tapered off. Routine chest tube that was placed perioperatively to drain excess mediastinal blood was removed the day after surgery. On postoperative day 1, a rhythm strip recorded 4 s of high‐grade atrioventricular block with ventricular standstill (VS) (Figure 2). The patient remained asymptomatic during the event, including being fully alert and oriented without prodrome of presyncope or syncope. Unfortunately, telemetry continued to record multiple episodes of VS leading into postoperative day 2, all of which ranged from 3 to 5 s. During these events, he did not have syncopal symptoms, nor was he ever paced by his pacer wires. Analysis of electrolytes showed potassium ranged from 3.6–4.1 mEq/L, sodium 134–140 mEq/L, serum calcium 7.8–8.4 mg/dL, and magnesium 2.1–2.5 mg/dL. On postoperative day 3, the rhythm strip recorded atrial flutter with loss of capture with loss of capture from the epicardial pacer (Figure S1). Once again, he remained asymptomatic and did not require pacing from his pacer wires.

## 5 DIFFERENTIAL DIAGNOSIS

Development of arrhythmias after CABG are a common occurrence and primarily include atrial fibrillation and atrial flutter. Ventricular tachyarrhythmias such as non‐sustained ventricular tachycardia are often seen, but usually transient and do not require procedural intervention. Conduction disturbances and bradyarrhythmias such as atrioventricular node block and sinus node dysfunction are the least common types of arrhythmias observed post‐CABG.

## 6 MANAGEMENT

**FIGURE 3:**
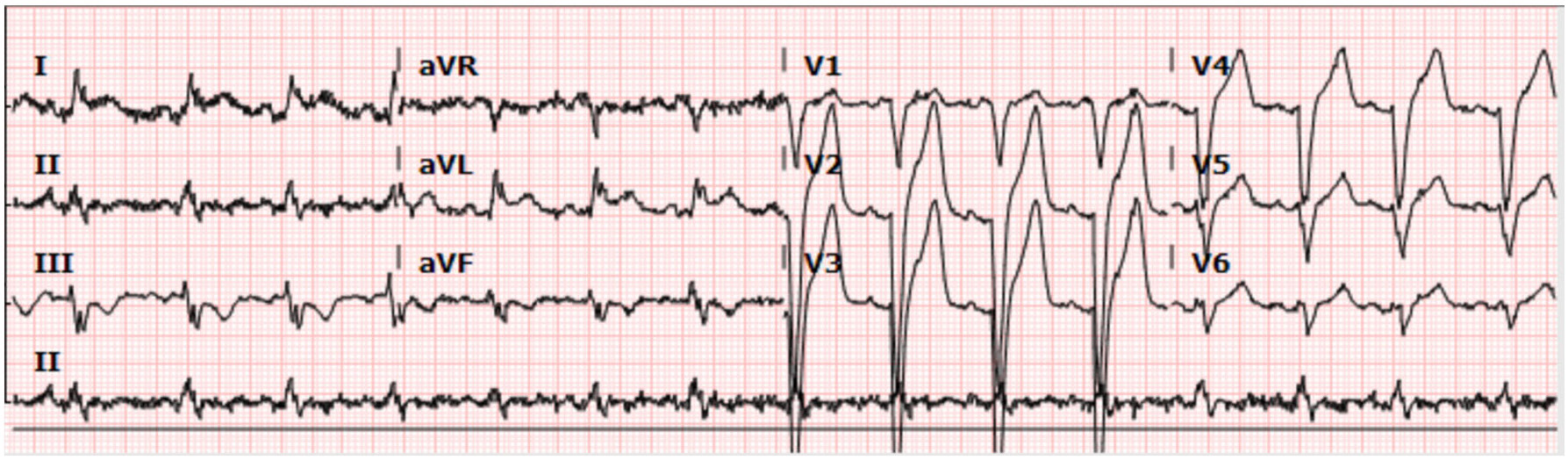
Postoperative day 5 EKG after implantation of dual chamber pacemaker. Patient is being ventricularly paced, although pacer spikes are not well visualized in this view.

Due to the patient's dependence on his temporary pacemaker in the setting of multiple documented episodes of ventricular standstill, electrophysiology was consulted for placement of a permanent pacemaker. On postoperative day 5, a dual‐chamber Boston Scientific pacemaker was successfully implanted without complications and a follow‐up EKG recorded a paced rhythm without any runs of VS (Figure 3). His electrolytes, specifically potassium and magnesium, were analyzed daily, and his rhythm was monitored on telemetry until discharge. He did not require electrolyte repletion, and telemetry did not detect any concerning atrial or ventricular arrhythmias postoperatively. Basal‐bolus insulin was administered to maintain his sugars between 140 and 180 as per hospital protocol. Arrangements were made for him to participate in cardiac rehabilitation therapy 1 month after discharge.

## 7 FOLLOW‐UP

The patient followed up in electrophysiology clinic where interrogation of his pacemaker did not reveal significant atrial nor ventricular events, no periods of undersensing, nor inappropriate pacing. An atrial and ventricular paced rate of less than 1% was recorded. He denied angina, cardiac palpitations, and syncopal episodes since his discharge.

## 8 DISCUSSION

Ventricular standstill is an uncommon arrhythmia that occurs when conduction through the ventricles is interrupted, while the sinoatrial node remains functional.^2^ For this reason, EKG displays p waves without succeeding QRS waves, as ventricular depolarization is inhibited. Prevalence and incidence remain unclear, and many cases are likely missed as out‐of‐hospital incidents are likely to result in sudden cardiac death (SCD) if the patient is not being paced. Rubin et al. evaluated incidence of ventricular arrhythmias after CABG and found 57% developed complex ventricular arrhythmias including large premature ventricular contraction (PVC) burden, ventricular tachycardia, and R on T phenomenon.^3^ No perioperative risk factors were identified that predisposed the subjects to the arrhythmias, all of whom had normal LVEF.

This case highlights a unique presentation of VS as the patient remained completely asymptomatic despite never being paced by his pacer wires. This arrhythmia is known to be ten times more dangerous than ventricular fibrillation, making rapid diagnosis and intervention crucial to prevent hemodynamic collapse and SCD.^4^ Underlying coronary artery disease (CAD) is evident in approximately 80% of SCD cases; however, 24‐h ambulatory electrocardiography is preferred for optimal risk stratification.^5^ Although non‐sustained ventricular arrhythmias can often be conservatively managed with continued telemetry monitoring and correction of underlying electrolyte deficiencies, affected patients with structural heart disease require stricter surveillance. Specifically, severe derangements in potassium and magnesium are established independent risk factors for lethal ventricular arrhythmias. Potassium has a role in maintaining myocardial electrical stability via the sodium‐potassium adenosine triphosphatase pump.^6^ Adrenergic stimulation of this pump during myocardial ischemia can deplete potassium levels and predispose patients to ventricular arrhythmias and SCD.^6^ Magnesium regulates the activity of the renal outer medullary potassium (ROMK) channel and is inversely proportional to the open ROMK channel pores.^7^ Hence, decreased intracellular magnesium levels cause more ROMK channels to open and lead to potassium wasting and the development of lethal arrhythmias.^7^ Despite the nonsustained paroxysmal pattern of VS in our patient, the fact that his arrhythmia repeatedly occurred while being continuously paced necessitated permanent pacemaker implantation.

To the best of our knowledge, asymptomatic VS following CABG has not been reported based on a comprehensive literature query of PubMed and Google Scholar. The majority of cases involved infectious etiology or profound vagal tone leading to bradyarrhythmia and eventually VS. Our literature review of published case reports in PubMed since 2000 identified 35 patients who developed VS (Table 1). Females accounted for 57% of cases and underlying structural heart disease was present in only 29% of patients. Myocarditis and excessive vagal tone were the two most common causes with each responsible for 20% of cases. Remarkably, only two patients (8%) were asymptomatic at the time VS was diagnosed during their hospital stay. Nine patients were excluded from this measurement due to being sedated on venoarterial extracorporeal membrane oxygenation (VA‐ECMO), mechanically ventilated, or symptoms were not documented in the manuscript. We postulate many cases of VS go unreported as more than seventy percent of cardiac arrests and subsequent SCDs precipitated by this ventricular arrhythmia occur outside of the hospital.^8^

**TABLE 1:** Characteristics of patients and etiologies of ventricular standstill based on a query of PubMed case reports since 2000.

Year	Age/Sex	Cause	Structural heart disease before presentation	Symptomatic during ventricular standstill	Citation
2023	46 y/o female	Fulminant HHV‐6 myocarditis	No	No‐intubated and sedated on VA‐ECMO	1. Golob S, Nazeer H, Kadosh B, et al. HHV‐6 myocarditis progressing to ventricular standstill requiring cardiac transplant. *JACC Case Report* 2023;17:101896–101896. doi: 10.1016/j.jaccas.2023.101896
2023	56 y/o male	Percutaneous coronary intervention	Yes‐Multivessel coronary disease	Yes	Takahashi K, Takemoto M, Sakaue T, Ikeda S, Okura T. Vasospasm in the first septal perforator branch and late high‐grade atrioventricular block following successful primary percutaneous coronary intervention for the proximal left anterior descending coronary artery: a case report. *Cureus*. 2023;15(5):e39172
2023	72 y/o female	Complication of aortic and mitral valve replacement	Yes‐Aortic stenosis, hypertrophic cardiomyopathy, and mitral regurgitation	Unspecified	Glines K, Hayanga JWA, Gibson C, El Churafa M, Wei L, Hayanga HK. Triple threat: significant concomitant aortic stenosis, mitral stenosis, and systolic anterior motion of the mitral valve causing left ventricular outflow tract obstruction in cardiac surgical patients. *Case Rep Anesthesiol*. 2023;2023:9995115
2023	46 y/o female	Fulminant myocarditis	No	Unspecified	Malamas P, Langione D, Nazeer H. Fulminant myocarditis in a middle‐aged woman with ulcerative colitis and recent exposure to covid‐19 requiring orthotopic heart transplanT. *J Am Coll Cardiol*. 2023;81(8):3586
2023	67 y/o male	Increased vagal tone (dizziness, presyncope, syncope)	Yes‐Unspecified heart disease	Yes	Seffah K, Agyeman WY, Cardona J, Berchie P. A deceptively unremarkable standstill: a case report of a rare cardiac electrophysiologic event. *Cureus*. 2023;15(1):e33763
2022	57 y/o male	Lymphocytic myocarditis	No	Yes	Kimball E, Buchwalder K, Upchurch C, Kea B. Intermittent complete heart block with ventricular standstill after Pfizer COVID‐19 booster vaccination: a case report. *J Am Coll Emerg Physicians Open*. 2022;3(2):e12723
2022	39 y/o female	Fulminant myocarditis	No	Unspecified	Thomson A, Totaro R, Cooper W, Dennis M. Fulminant delta COVID‐19 myocarditis: a case report of fatal primary cardiac dysfunction. *Eur Heart J Case Rep*. 2022;6(4):ytac142
2021	18 y/o male	PRKAG2 gene mutation	No	Yes	Navani RV, Koh Y, Voskoboinik A. Syncope in a young male. *Eur Heart J Case Rep*. 2021;5(11):ytab458
2021	38 y/o female	Fulminant myocarditis	No	No‐ sedated on ECMO	Lim Y, Kim MC, Kim KH, et al. Case report: acute fulminant myocarditis and cardiogenic shock after messenger RNA coronavirus disease 2019 vaccination requiring extracorporeal cardiopulmonary resuscitation. *Front Cardiovasc Med*. 2021;8:758996.
2021	70 y/o male	Increased vagal tone (presyncopal episode)	Yes‐Aortic stenosis	Yes	Moles WJ, Barnes AA, Khan A, Patel K, Bos N. Incidental findings of asystole in a patient with complaints of near syncope: a case report on paroxysmal ventricular standstill. *Cureus*. 2021;13(10):e18438.
2021	41 y/o female	Progression of high‐grade atrioventricular block	Yes‐Hypertrophic obstructive cardiomyopathy	Yes	Mistry A, Assuvinkumar S, Gador G, Somani R. Atrioventricular synchronous pacing using leadless pacemaker in a heart transplant patient. *BMJ Case Rep*. 2021;14(6):e243365
2021	53 y/o female	Mediastinal radiotherapy for Non‐Hodgkin's lymphoma	No	Yes	Ali S, Ali O, Ahmed I, Nazir T. Trifascicular block and ventricular standstill: a late complication of mediastinal radiotherapy in a cancer survivor. *Cureus*. 2021;13(1):e12806
2021	55 y/o female	POEMS syndrome	No	No‐ mechanically ventilated	Tan JH, Yew MS, Huang W, Tan K. Left ventricular systolic dysfunction with concomitant bradyarrhythmia in a patient with POEMS syndrome: a case report. *Eur Heart J Case Rep*. 2021;5(2):ytaa510
2020	40 y/o male	Stokes–Adams syndrome	No	Yes	Adegoke DA. Paroxysmal ventricular standstill: a rare cardiac manifestation of syncope. *Am J Case Rep*. 2020;21:e924381
2020	59 y/o male	Acute gastrointestinal bleed	No	Yes	Latt H, Kyaw K, Tun NM, Tun TT, Aung SSM, Yin HH. A case of ventricular standstill in a patient with acute gastrointestinal bleeding. *J Community Hosp Intern Med Perspect*. 2020;10(3):283–286
2019	82 y/o female	Transcatheter aortic valve implantation	Yes‐Severe aortic stenosis, non‐obstructive CAD	Yes	Wheen P, Armstrong R, Maree A, O'Connor S. Late ventricular standstill following an elective TAVI. *BMJ Case Rep*. 2019;12(12):e232477
2018	64 y/o female	Blunt chest trauma from motor vehicle accident	No	No	Soud M, Alrifai A, Kabach A, Fanari Z, Alraies MC. Trauma‐induced conduction disturbances. *Ochsner J*. 2018;18(3):277–279
2018	24 y/o 6‐month postpartum female	Takotsubo cardiomyopathy	No	Yes	Lee N, Lee KW, D'Ambrosio MM, Banta JV, Voudouris A, Tsompanidis A. Takotsubo syndrome‐associated ventricular standstill in a peripartum patient: case report and review of the literature. *Clin Case Rep*. 2017;6(2):283–287
2018	36 y/o male	Fulminant myocarditis	No	No‐ sedated on VA‐ECMO	Segawa T, Arita Y, Akari T, Hasegawa S. Fulminant myocarditis. *BMJ Case Rep*. 2018;2018:bcr2017223973
2016	66 y/o female	Takotsubo cardiomyopathy	No	Yes	Gamble DT, Shuttleworth KJ, Scally C, Leslie SJ. Takotsubo cardiomyopathy with severe bradyarrhythmia following epidural insertion. *BMJ Case Rep*. 2016;2016:bcr201621694
2016	33 y/o male	Complication of closure of truncus arteriosus	Yes‐Truncus arteriosus	Unspecified	Ruan W, Loh YJ, Guo KW, Tan JL. Surgical correction of persistent truncus arteriosus on a 33‐year‐old male with unilateral pulmonary hypertension from migration of pulmonary artery band. *J Cardiothorac Surg*. 2016;11:39
2016	49 y/o female	Intravenous erythromycin	No	No	Khan S, Ramzy J, Papachristos D, George N, Fisher L. Ventricular standstill following intravenous erythromycin and borderline hypokalemia. *Eur J Case Rep Intern Med*. 2016;3(3):000375
2014	23 y/o pregnant female	Increased vagal tone (palpitations, dizziness, and presyncope)	No	Yes	Sengupta A, Slater TA, Sainsbury PA. The investigation and management of broad complex tachycardia and ventricular standstill presenting in pregnancy: A case report. *Obstet Med*. 2014;7(3):131–134
2014	50 y/o female	Increased vagal tone from nausea/vomiting and rapid eye movement sleep	Yes‐Membranous ventricular septal defect, non‐obstructive CAD	Yes	Jaiswal S, Aldave AP, Wool KJ. Ventricular standstill: An uncommon electrophysiological abnormality caused by profound vagal tone. *N Am J Med Sci*. 2014;6(4):178–180
2014	14‐month‐old male	Lower respiratory tract infection progressing to septic shock	No	Yes	Lynch RM, Ballesty L, Maicoo R. “Be still my beating heart”: Ventricular standstill occurring in different age groups. *African Journal of Emergency Medicine*. 2014;4(4):e12‐e15. doi:10.1016/j.afjem.2014.02.005
2014	19 y/o non‐pregnant female	Increased vagal tone (dizziness + presyncopal prodrome)	No	Yes	Lynch RM, Ballesty L, Maicoo R. “Be still my beating heart”: Ventricular standstill occurring in different age groups. *African Journal of Emergency Medicine*. 2014;4(4):e12‐e15. doi:10.1016/j.afjem.2014.02.005
2014	50 y/o female	Increased vagal tone (multiple syncopal episodes + sinus bradycardia 30 bpm)	No	Yes	Lynch RM, Ballesty L, Maicoo R. “Be still my beating heart”: Ventricular standstill occurring in different age groups. *African Journal of Emergency Medicine*. 2014;4(4):e12‐e15. doi:10.1016/j.afjem.2014.02.005
2012	46 y/o male	Transcatheter aortic valve replacement	Yes‐Transposition of great arteries, atrial and ventricular septal defects, situs inversus, and pulmonary outflow tract stenosis	Unspecified	Hamilton P, Coverdale A, Edwards C, et al. Transcatheter aortic valve implantation in end‐stage renal disease. *Clin Kidney J*. 2012;5(3):247–249
2012	60 y/o male	Increased vagal tone (intermittent dizziness spells)	No	Yes	Sidhu M, Singh HP, Chopra AK, Kapila D, Sidhu S. Surviving ventricular standstill for 111 seconds during Holter monitoring. *Ann Noninvasive Electrocardiol*. 2012;17(1):61–62
2011	36 y/o female	Wegener's granulomatosis	No	Yes	Cassidy CJ, Sowden E, Brockbank J, Teh LS, Ho E. A patient with Wegener's granulomatosis in apparent remission presenting with complete atrioventricular block. *J Cardiol Cases*. 2011;3(2):e71‐e74
2010	62 y/o male	Dual chamber pacemaker malfunction	No‐pacemaker was placed for atrioventricular block due to myocarditis	Yes	van de Sandt FM, Jansen R, Kimman GP, Ruiter JH. Ventricular asystole due to inhibition of ventricular backup pacing by a dual chamber pacemaker: possible pitfalls of default settings. *Neth Heart J*. 2010;18(6):323–326
2007	3‐week‐old infant	Short QT syndrome	No	Yes	Morphet JA. The short QT syndrome and sudden infant death syndrome. *Can J Cardiol*. 2007;23(2):105
2006	11 y/o female	Complication of closure of perimembranous ventricular septal defect	Yes‐perimembranous septal defect	Yes	Walsh MA, Bialkowski J, Szkutnik M, Pawelec‐Wojtalik M, Bobkowski W, Walsh KP. Atrioventricular block after transcatheter closure of perimembranous ventricular septal defects. *Heart*. 2006;92(9):1295–1297
2005	82 y/o male	Intracerebral hematoma	No	Yes	Mandal AK, Baltsezak S, Missouris CG. Intracerebral haematoma masquerading as ventricular standstill. *Heart*. 2005;91(1):e1
2002	5 y/o female	Myocarditis	No	Yes	Leonard PA, Burns JE. Failure to recognise ventricular standstill. *Emerg Med J*. 2002;19(1):86–87

## 9 CONCLUSIONS

Ventricular standstill is a dangerous arrhythmia that requires prompt diagnosis and intervention, especially in patients with structural heart disease who are already at heightened risk for adverse cardiac events. Clinicians should recognize VS as a potential complication of cardiac revascularization and be cognizant of asymptomatic cases necessitating intervention. Early evaluation by electrophysiology to facilitate pacemaker implantation portends good outcomes in this patient subgroup.

## 10 LEARNING OBJECTIVES

- To recognize ventricular standstill as a potential complication of cardiac revascularization.
- To emphasize syncopal symptoms do not need to be present in non‐sustained ventricular standstill to warrant pacemaker implantation.

## AUTHOR CONTRIBUTIONS

**Andrew Sagalov:** Conceptualization; writing – original draft; writing – review and editing. **Shashi Maryala:** Writing – review and editing. **Abhishek Kulkarni:** Supervision; validation. **Shruti Hegde:** Supervision; validation. **Mohamed Labedi:** Supervision; validation; writing – review and editing.

## FUNDING INFORMATION

We have no funding sources to report.

## CONFLICT OF INTEREST STATEMENT

None of the authors have any industry relationships nor conflicts of interest, financial or otherwise, to report.

## CONSENT

With the exception of age and sex, no identifying information was used in the manuscript. Written informed consent was obtained from the patient for the use of their hospital course and imaging in accordance with the journal's patient consent policy.

## Supporting information


Figure S1.

Video S1.

Video S2.

## Data Availability

Data sharing is not applicable to this article as no datasets were generated or analyzed during the current study.

## References

[1] Vos RJ , Jongbloed L , Sonker U , Kloppenburg GTL . Titanium plate fixation versus conventional closure for sternal dehiscence after cardiac surgery. Thorac Cardiovasc Surg. 2017;65(4):338‐342. 27177262 10.1055/s-0036-1583297

[2] Jaiswal S , Aldave AP , Wool KJ . Ventricular standstill: an uncommon electrophysiological abnormality caused by profound vagal tone. N Am J Med Sci. 2014;6(4):178‐180. 24843851 10.4103/1947-2714.131245 PMC4024585

[3] Rubin DA , Nieminski KE , Monteferrante JC , Magee T , Reed GE , Herman MV . Ventricular arrhythmias after coronary artery bypass graft surgery: incidence, risk factors and long‐term prognosis. J Am Coll Cardiol. 1985;6(2):307‐310. 3874891 10.1016/s0735-1097(85)80165-0

[4] Phibbs B . The Human Heart: A Basic Guide to Heart Disease. 2nd ed. Lippincott Williams and Wilkins; 2007:111‐112.

[5] Myerburg RJ , Interian A Jr , Mitrani RM , Kessler KM , Castellanos A . Frequency of sudden cardiac death and profiles of risk. Am J Cardiol. 1997;80(5B):10F‐19F. 10.1016/s0002-9149(97)00477-3 9291445

[6] Ozen Y , Ozbay MB , Ertem AG , Yayla Ç . Serum electrolyte levels and ventricular arrhythmia. Angiology. 2019;70(1):87‐88. 30114942 10.1177/0003319718794573

[7] Negru AG , Pastorcici A , Crisan S , Cismaru G , Popescu FG , Luca CT . The role of hypomagnesemia in cardiac arrhythmias: a clinical perspective. Biomedicine. 2022;10(10):2‐5. 10.3390/biomedicines10102356 PMC9598104 36289616

[8] Tsao CW , Aday AW , Almarzooq ZI , et al. Heart disease and stroke Statistics‐2022 update: a report from the American Heart Association [published correction appears in circulation. 2022 Sep 6;146(10):e141]. Circulation. 2022;145(8):e153‐e639. 35078371 10.1161/CIR.0000000000001052

