# Supplementary material for: Asymptomatic paroxysmal ventricular standstill in an elderly male following revascularization of multivessel coronary disease: A case report and literature review
Source: Clin Case Rep. 2024 Mar 21;12(3):e8675. doi: 10.1002/ccr3.8675 (PMC10957490; doi:10.1002/ccr3.8675)

**Supplemental Appendix**

**Video 1:** Left heart catheterization images showing multi-vessel obstructive coronary disease including a 60% lesion proximal to bifurcation of left anterior descending artery and circumflex artery, severe diffuse disease in first obtuse marginal artery, and a 30-40% lesion in ostium of the right posterior descending artery.

**Video 2:** Limited echocardiogram showing a left ventricular ejection fraction of 40-45% and hypokinetic changes in the anterior and lateral walls.

**Supplementary Figure S1:** Postoperative day three rhythm strip showing atrial flutter with classic sawtooth waves and loss of capture from the pacer in lead II.


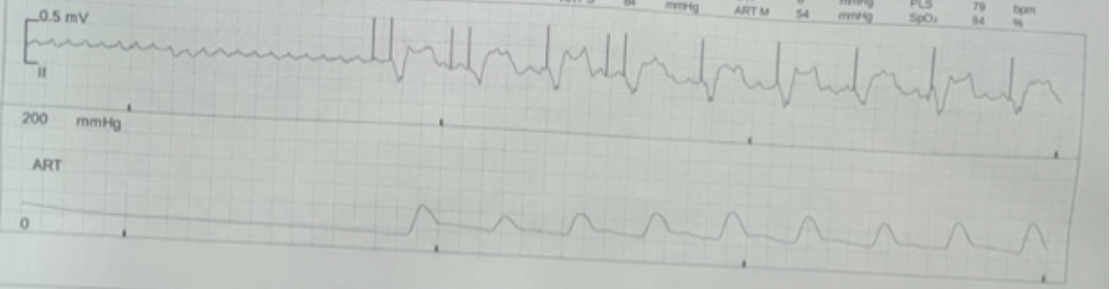

Supplement: Supplementary file 1 — Figure S1. [file CCR3-12-e8675-s002.docx]
